# T Cell Receptor-Independent, CD31/IL-17A-Driven Inflammatory Axis Shapes Synovitis in Juvenile Idiopathic Arthritis

**DOI:** 10.3389/fimmu.2018.01802

**Published:** 2018-08-06

**Authors:** Ian D. Ferguson, Patricia Griffin, Joshua J. Michel, Hiroshi Yano, Sarah L. Gaffen, Robert G. Mueller, Jeffrey A. Dvergsten, Jon D. Piganelli, Margalit E. Rosenkranz, Daniel A. Kietz, Abbe N. Vallejo

**Affiliations:** ^1^Department of Pediatrics, University of Pittsburgh, Pittsburgh, PA, United States; ^2^Children’s Hospital of Pittsburgh, University of Pittsburgh Medical Center, Pittsburgh, PA, United States; ^3^Graduate Program in Microbiology and Immunology School of Medicine, University of Pittsburgh, Pittsburgh, PA, United States; ^4^Department of Medicine, Division of Rheumatology and Clinical Immunology, University of Pittsburgh, Pittsburgh, PA, United States; ^5^Department of Immunology, University of Pittsburgh, Pittsburgh, PA, United States; ^6^Department of Pediatrics, Duke University Medical Center, Durham, NC, United States; ^7^Department of Surgery, University of Pittsburgh, Pittsburgh, PA, United States

**Keywords:** CD31, double negative alpha beta T cells, fibrocyte-like cells, IL-17, juvenile idiopathic arthritis, oxidoreductase, synovial inflammation, TCR-independent

## Abstract

T cells are considered autoimmune effectors in juvenile idiopathic arthritis (JIA), but the antigenic cause of arthritis remains elusive. Since T cells comprise a significant proportion of joint-infiltrating cells, we examined whether the environment in the joint could be shaped through the inflammatory activation by T cells that is independent of conventional TCR signaling. We focused on the analysis of synovial fluid (SF) collected from children with oligoarticular and rheumatoid factor-negative polyarticular JIA. Cytokine profiling of SF showed dominance of five molecules including IL-17A. Cytometric analysis of the same SF samples showed enrichment of αβT cells that lacked both CD4 and CD8 co-receptors [herein called double negative (DN) T cells] and also lacked the CD28 costimulatory receptor. However, these synovial αβT cells expressed high levels of CD31, an adhesion molecule that is normally employed by granulocytes when they transit to sites of injury. In receptor crosslinking assays, ligation of CD31 alone on synovial CD28^null^CD31^+^ DN αβT cells effectively and sufficiently induced phosphorylation of signaling substrates and increased intracytoplasmic stores of cytokines including IL-17A. CD31 ligation was also sufficient to induce RORγT expression and *trans*-activation of the *IL-17A* promoter. In addition to T cells, SF contained fibrocyte-like cells (FLC) expressing IL-17 receptor A (IL-17RA) and CD38, a known ligand for CD31. Stimulation of FLC with IL-17A led to CD38 upregulation, and to production of cytokines and tissue-destructive molecules. Addition of an oxidoreductase analog to the bioassays suppressed the CD31-driven IL-17A production by T cells. It also suppressed the downstream IL-17A-mediated production of effectors by FLC. The levels of suppression of FLC effector activities by the oxidoreductase analog were comparable to those seen with corticosteroid and/or biologic inhibitors to IL-6 and TNFα. Collectively, our data suggest that activation of a CD31-driven, αβTCR-independent, IL-17A-mediated T cell-FLC inflammatory circuit drives and/or perpetuates synovitis. With the notable finding that the oxidoreductase mimic suppresses the effector activities of synovial CD31^+^CD28^null^ αβT cells and IL-17RA^+^CD38^+^ FLC, this small molecule could be used to probe further the intricacies of this inflammatory circuit. Such bioactivities of this small molecule also provide rationale for new translational avenue(s) to potentially modulate JIA synovitis.

## Introduction

Juvenile idiopathic arthritis (JIA) is a highly prevalent rheumatic disease of childhood before the age of 16 years. It is a genetic and clinical entity distinct from adult-onset rheumatoid arthritis (RA). The clinical subtypes of JIA have their own treatment patterns and prognostic courses (1). Thus, elucidating immunopathways unique to JIA may be informative for therapeutic innovations.

Multiple HLA associations of JIA implicate involvement of T cells (2). Oligoclonal expansions of these cells in blood and synovial fluid (SF) are well documented (3). However, the antigenic driver(s) of oligoclonality, or antigenic cause(s) of JIA disease remain elusive. Nevertheless, T cells play a role in JIA pathogenesis as they comprise a significant proportion of joint-infiltrating cells (4, 5).

Early studies on T cells in JIA showed many cells lacked expression of the costimulatory molecule CD28 (6). We have reported that there is a pervasive CD28^null^CD8^+^ T cell population in oligoarticular and rheumatoid factor (RF)-negative polyarticular JIA (7), the two most prevalent clinical subtypes. CD28 is required to sustain T cell activation, but is irreversibly lost with chronologic aging (8). Aged human CD28^null^ CD4^+^ and CD8^+^ T cells are nonetheless functionally active (9), due in part to *de novo* expression of other molecules such as NK-related receptors CD56 and NKG2D that are capable of directly activating T cells (10). In JIA, we reported the *in vivo* accumulation of CD28^null^CD8^+^ T cells disproportionately with age (7). This CD8 subset is prematurely senescent as indicated by their shortened telomeres, limited proliferative capacity, and expression of mitotic inhibitors. Furthermore, they express CD31, a receptor normally employed by granulocytes during their entry into sites of injury (11). In mice, *CD31*^−/−^ granulocytes are unable to traverse endothelial barriers (12). Thus, accumulation of CD31^+^CD28^null^CD8^+^ T cells in JIA SF suggests their pathogenic role.

We sought to further evaluate the functional relevance of CD31 expression on joint-infiltrating T cells. We analyzed SF from another JIA cohort. Here, we report a subset of CD31^+^CD28^null^ αβT cells that lack expression of both CD4 and CD8, referred to as double negative (DN) T cells. We hypothesized that CD31-driven T cell activation elaborates an inflammatory signature of SF. Of interest is whether synovial CD31^+^CD28^null^ DN and CD8^+^ αβT cells respond similarly, or differently, to CD31 triggering. A corollary hypothesis is whether molecular effector(s) derived from CD31-activated synovial αβT cells leads to downstream activation of other SF mononuclear cells (SFMC) thereby compounding local inflammation.

## Materials and Methods

### Human Subjects and Biological Specimens

Institutional Review Boards of the University of Pittsburgh approved all research protocols. Written informed consent from parents/legal guardians, and assent of child subjects as appropriate, were obtained. Children with oligoarticular or RF^−^ polyarticular with JIA were recruited from the Rheumatology Clinic of Children’s Hospital of Pittsburgh. RF^+^ polyarthritis, enthesitis-related arthritis, and psoriatic arthritis were excluded since these are considered separate genetic and clinical entities (13, 14). Patients undergoing arthrocentesis were targeted as donors of SF. Blood were also collected from our broader JIA patient base. Similar blood samples were collected from healthy controls, or those de-identified waste/residual clinical samples from our Clinical Laboratory Services.

As we have done in previous studies (7, 15–17), cell-free plasma was prepared by low speed centrifugation of blood. Similar centrifugation was carried out to isolate cell-free SF preparations from whole SF. Blood and SF samples with evidence of hemolysis were not used for these cell-free preparations. The centrifuged blood and SF samples were subsequently used to isolate peripheral blood mononuclear cells (PBMC) and SFMC, respectively, by standard isopycnic centrifugation. Cell contamination of plasma/SF preparations, and cell viability of PBMC/SFMC were verified by trypan blue staining, and/or by live cell counting using Countess II (Life Technologies). Aliquots of cell-free plasma/SF were stored in −80°C freezer until analysis. PBMC and SFMC aliquots were cryopreserved using a standardized protocol that we had validated previously to yield >90% recovery (7, 16).

### Flow Cytometry

Multicolor cytometry was performed on SFMC and PBMC stained with fluorochrome-conjugated antibodies to cell surface and intracellular markers. All samples included a cell viability dye (ZombieUV™, BioLegend), which was used for the electronic gating of live cells. Raw cytometry data were acquired using a custom 5-laser Aria II cytometer (BD Biosciences). Off-life analyses of cell populations were performed using FlowJo software (Tree Star). Immunostaining, instrument calibration, signal optimization, and off-line analyses employed our standardized procedures (7, 16).

We focused on conventional T cells defined as TCRαβ^+^ (T10B9, BD), and were examined for expression of CD4 (OKT4, BD), CD8 (RPA-T8, BioLegend), CD28 (CD28.2, Novus Biologicals), and CD31 (2H8, Abcam). Discrimination of αβT cell subsets was achieved by exclusion staining for γδT cells (5A6.E91, ThermoFisher), monocytes (CD14: M5E2, BD), B cells (CD19: SJ25-C1, BD), NK cells (CD16/CD56: 3G8/NCAM16.2, BD), and plasma cells (CD138: 281-2, BD).

Non-lymphoid SFMC were also screened for co-expression of procollagen 1 (2Q576, Abcam) and proline-4-hydroxylase (EPR3661, Abcam), markers of mesenchymal fibrocytes reportedly found in circulation especially during injury (18). Since these markers are also characteristic of tissue fibroblasts (19, 20), we coined the term “fibrocyte-like cells (FLC)” reflecting their yet undetermined origin. We employed an immunostaining protocol for FLC by excluding the above mentioned hematopoietic markers and also tissue macrophages (CD68: Y1/82A, BD) in a single channel. For FLC, we screened for expression of IL-17 receptor A (IL-17RA) (0A01905, BD) and CD38 (HIT2, BioLegend).

### Multiplex Analyses of Cell-Free SF and Plasma

A panel of 25 cytokines/chemokines was examined based on a broader global cytokine screening reported by de Jager et al. (21) and from our survey of current literature about their relevance to inflammation in general, and in disease settings of non-infectious arthritis. Using a customized kit (MILLIPLEX^®^ MAP, Millipore Sigma) cytokine analyses were performed using previously standardized Luminex procedures (7). Raw data were acquired using MAGPIX or BioPlex200 (BioRad). Data quality was ascertained by a standard curve for each plate. We routinely set two overlapping standard curves, above and below the manufacturer’s recommended setting. These plate curves were then used to determine confidence intervals in the construction a normalization curve. The latter was then used to adjust intra-plate variations and to calculate cytokine concentrations.

### T Cell Bioassays

Preparation of cells for intracellular cytometry followed previous procedures (7, 16). CD31^+^CD28^null^ DN and CD8 αβT cells were enriched from SFMC using EasySep (Stem Cell Technologies); preparations >85% enrichment used in experiments. Jurkat and JRT3 (both purchased from ATCC) were used as cell models for CD31^+^CD3^+^TCR^+^, and CD31^+^CD3^−^TCR^−^, respectively; these phenotypes verified by cytometry. Receptor crosslinking was performed according to previous procedures (7) using anti-CD31 (WM59, ab218, Abcam), anti-CD3 (OKT3, Centocor Ortho Biotech), anti-TCRαβ (1P26, BioLegend), or normal mouse Ig (BD) as stimulators. Crosslinking was achieved by excess amount of Cy5-conjugated anti-mouse Ig (BioLegend). Cytometry was performed by gating on cross-linked αβT cells, visualized as Cy5^+^ cells that were also CD8^+^CD4^−^ or CD4^−^CD8^−^.

For some cultures, two small molecule inhibitors were added. One is Imatinib (Gleevec^®^, Selleck Chem), an inhibitor of catalytically active cAbl kinase that is currently used in the treatment of chronic myelogenous leukemia (CML) (22). It was added at 50 nM. The other is Mn(III) meso-tetrakis(N-ethylpyridinium-2-yl)porphyrin (MnT2E), a Mn porphyrin mimic of superoxide dismutase that was originally developed to neutralize the tissue-destructive effects of superoxide (23). It has been shown experimentally to also inhibit the DNA-binding activity of NFκB (24). It was added at 34 µM (GMP grade, provided by Albany Molecular Research Inc., to JDP). The concentrations of Imatinib and MnT2E used were empirically determined as non-toxic (>95% cell viability).

For intracellular detection of IL-6 (AS12, BD), IL-17A (BL168, BioLegend), TNFα (Mab11, BD), and IFNγ (B27, BD), and RORγT (Q21-559, BD), a regulator *IL-17A* gene transcription (25), the crosslinked cells were cultured for 6 h in the presence of GolgiPlug™ reagent (BD) (7) in 7.5% CO_2_ at 37°C. For signaling intermediates, the phosphorylated forms of ZAP70 (Y272; J34-602, BD), serine-threonine kinase Akt (S473; M89-61, BD), p16 subunit of NFκB referred to as RelA (S529; K10-895.12.50, BD), and Abelson kinase cAbl (Y245; ab62189, Abcam) were examined within 10 min of receptor crosslinking. These signaling phosphoproteins were identified from empirical proteomic screening (Hypromatrix). All intracellular cytometry procedures were performed according to our previous protocols (7).

### Confocal Microscopy

Cells were incubated with anti-CD31 as described above. This was followed by crosslinking with anti-IgG immobilized onto microbeads labeled with Allophycocyanin (Spherotec). After 10 min, cells were fixed in paraformaldehyde, permeabilized with 0.1% Triton-PBS, washed, and blocked in 20% donkey serum. Cells were then incubated for 18 h with anti-phospho-Y245 cAbl (ab62189, Abcam) at 4°C, followed by anti-IgG conjugated with fluorescein isothiocyanate (Abcam) for 2 h at room temperature, counterstained with 4′,6-diamidino-2-phenylindole (Invitrogen), and applied to a glass coverslip with Aqua PolyMount. Images were acquired on an Fluoview 1000 confocal microscope (Olympus).

### FLC Bioassays

SFMC were first cultured overnight. The plastic-adherent cells were expanded to >70% confluence. Purity of the cultures determined cytometrically. FLC between second and fifth passages were incubated with or without non-toxic 20–2,000 ng/ml recombinant IL-17A (R&D Systems). In other experiments, FLC were cultured in 200 ng/ml IL-17A with the addition of 5 µM of a corticosteroid (Triamcinolone Acetonide, Aristospan^®^) or the biologic inhibitor of TNF (TNFi) Infliximab (Remicade^®^), or the biologic inhibitor of IL-6 (IL6i) Tocilizumab (Actemra^®^); or 34 µM MnT2E. After 24 h, CD38 expression was measured cytometrically, and the types and concentrations of soluble factors in the culture supernatant were examined by Luminex using a kit (LXSAHM18, R&D Systems). This kit consists of 18 molecules based on the global SF screening of de Jager et al. (21) and reports about IL-17A-induced molecules in other experimental systems including adult arthritis (26–29).

### Transient Transfection

With their homogeneous phenotype, Jurkat and JRT3 were used to test specifically the CD31-driven induction of IL-17A. Twenty µg luciferase plasmid reporter controlled by full-length *IL-17A* gene promoter (30), and 20 ng pRL *Renilla* luciferase plasmid (Promega) were co-transfected into 1 × 10^6^ cells using Lipofectamine (ThermoFisher). Subsequently, receptor crosslinking was performed as described above. As system control, transfected cells were also stimulated with phorbol myristyl acetate (PMA) and ionomycin. Normalized luciferase reporter activity was determined as described previously (30).

### Statistical Analysis

Data analyses were performed using SPSS software (V24, IBM). Due to intrinsic individual variations, data from T cell and FLC bioassays were normalized by expressing each response as stimulation index, or as percent (or fold) induction above or below the media or IgG controls as we have done previously (7). Stimulation indices were calculated from the difference of the experimental value and the media control divided by the appropriate IgG isotype control or solvent/carrier media as in the case of bioassays with Imatinib and MnT2E. We used this procedure reproducibly in a variety of experimental settings (7, 9, 16, 31–33). Kruskal–Wallis analysis of variance (ANOVA) was performed and *post hoc* pair-wise comparisons used the Tukey statistic. *P*-value <0.05 was considered significant.

## Results

### Characteristics of the Study Cohort

Consistent with epidemiologic studies (34) JIA patients examined were predominantly female as shown in Table 1. This gender-bias was used as reference for the random subsampling of an equivalent female-biased healthy group. The entire cohort was predominantly Caucasian, representative of our patient population. Patients had long-standing oligoarticular or RF^−^ polyarticular disease. They had varying age of disease onset and disease duration. There were no blood and SF samples from the same patient. Donors of SF were slightly older. There were multiple medications used, including topical steroids mirroring the patients with confirmed uveitis. None of the patients were newly diagnosed cases. Although anti-nuclear antibody (ANA) serology data were not available for all patients, about half of those tested were ANA^+^. At the time of consent/assent, patients had 1–3 swollen large joints.

**Table 1:** Characteristics of study cohort^a^.

	Healthy	Oligoarticular juvenile idiopathic arthritis (JIA)		Rheumatoid factor^**−**^ polyarticular JIA	
Specimen type	Blood	Blood	Synovial fluid (SF)	Blood	SF
Number of samples (# Black)	30 (4)	32	39 (2)	30	15
Sex, female/male	17/13	20/12	25/14	18/12	9/6
Mean age, years
Boys					
At sampling	8.99	9.43	11.37	10.7	16.26
At disease onset	(n/a)	6.96	9.55	5.84	8.19
Girls					
At sampling	12.7	9.51	10.12	10.7	16.03
At disease onset	(n/a)	6.37	6.64	6.4	5.7
Mean of disease duration (years)	(n/a)	3.62	2.71	4.5	10.44
Medication^b^
NSAID	(n/a)	25	25	16	9
Steroids, oral	(n/a)	2	1	2	1
Steroids, IA (total joints injected)	(n/a)	10 (20)	39 (62)	9 (22)	15 (18)
Steroids, IV	(n/a)	0	0	0	0
Steroids, topical	(n/a)	0	6	0	1
Methotrexate	(n/a)	12	7	19	5
Biologic agents	(n/a)	4	1	8	3
Other DMARD	(n/a)	2	2	2	3
Anti-nuclear antibody^+^ (number subjects tested)	(n/a)	16 (30)	15 (38)	7 (19)	5 (15)
Number of involved joints at sampling	(n/a)	1.03	1.53	2.27	1.83
Uveitis history	(n/a)	0	6	0	1

*^a^Blood and SF aspirates were obtained at during routine medical visits of patients. There were no paired blood-SF samples*.

*^b^Medications were tallied at the time of consent/assent. Some patients were taking multiple medications. NSAID: meloxicam, ibuprofen, tolmentin, naproxen, diclofenac, and indomethacin. Biologic agents: etanercept, adalimumab, and infliximab. Other DMARD: sulfasalazine and hydroxychloroquine*.

*(n/a), not applicable; DMARD, disease modifying anti-arthritic drug; IA, intraarticular injection; IV, intravenous injection; NSAID, non-steroidal anti-inflammatory drug*.

### Predominance of Five Cytokines in SF

Cytokines are recognized effectors of inflammation in JIA and have become therapeutic targets for neutralization using specific antibody blockers (35). Here, our primary goal was to examine the microenvironment of the JIA joint. Accordingly, experiments focused on SF, using PBMC and plasma from healthy controls and patients as internal references for SFMC phenotyping and cytokine profiling of cell-free SF, respectively.

**Figure 1:**
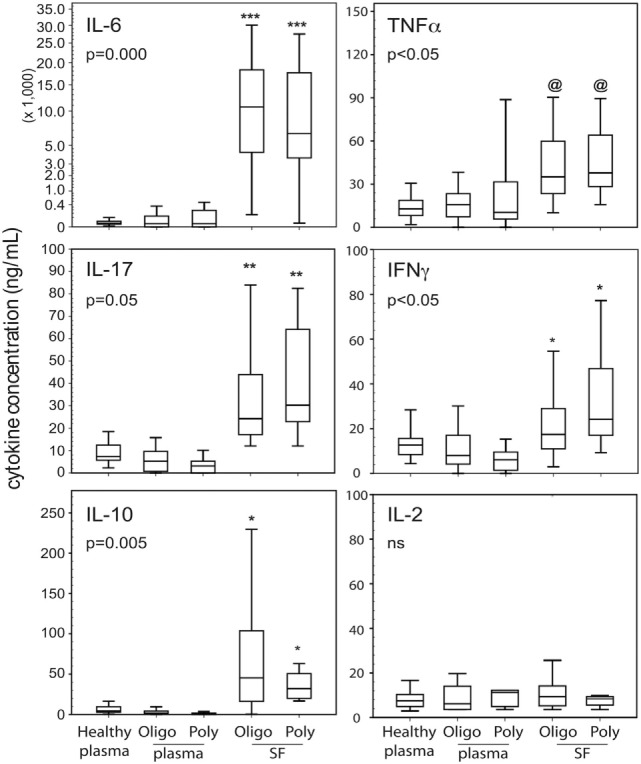
Cytokine profiles in blood and cell-free SF of JIA patients. Data shown (*n* = 15–39 per group as in Table 1) are box-median-whisker plots of the five most dominant cytokines found in SF compared to plasma. The boxes represent the 25th and 75th percentile of values. The whiskers were the 5th and 95th percentile of values. There was not a global cytokine upregulation as shown by negligible detection of IL-2. The indicated *P*-values were determined by Kruskal–Wallis ANOVA. Between group comparisons were made using the *post hoc* Tukey statistic: [***], [**], and [*] *P* < 0.005, *P* < 0.01, and *P* < 0.05, respectively, indicate JIA SF is significantly different from healthy and JIA plasma; [@] indicates polyarticular [poly]/oligoarticular [oligo] SF were not significantly different from poly JIA plasma, but were significantly different (*P* < 0.05) from oligo JIA and healthy plasma. Abbreviation: NS, not significant.

Of 25 cytokines and chemokines examined, Figure 1 shows IL-6, IL-10, IL-17A, IFNγ, and TNFα were found at significantly higher concentrations in SF compared to plasma. The cytokine SF/plasma levels of the two patient groups were equivalent. However, plasma levels of these five cytokines were not significantly different between patients and controls. Plasma cytokine levels were generally low. As depicted, IL-2 levels were not different between the subject groups thereby establishing confidence of the multiplex assay.

### CD31^+^CD28^null^ DN αβ T Cells Constitute a Major Cellular Component of SF

**Figure 2:**
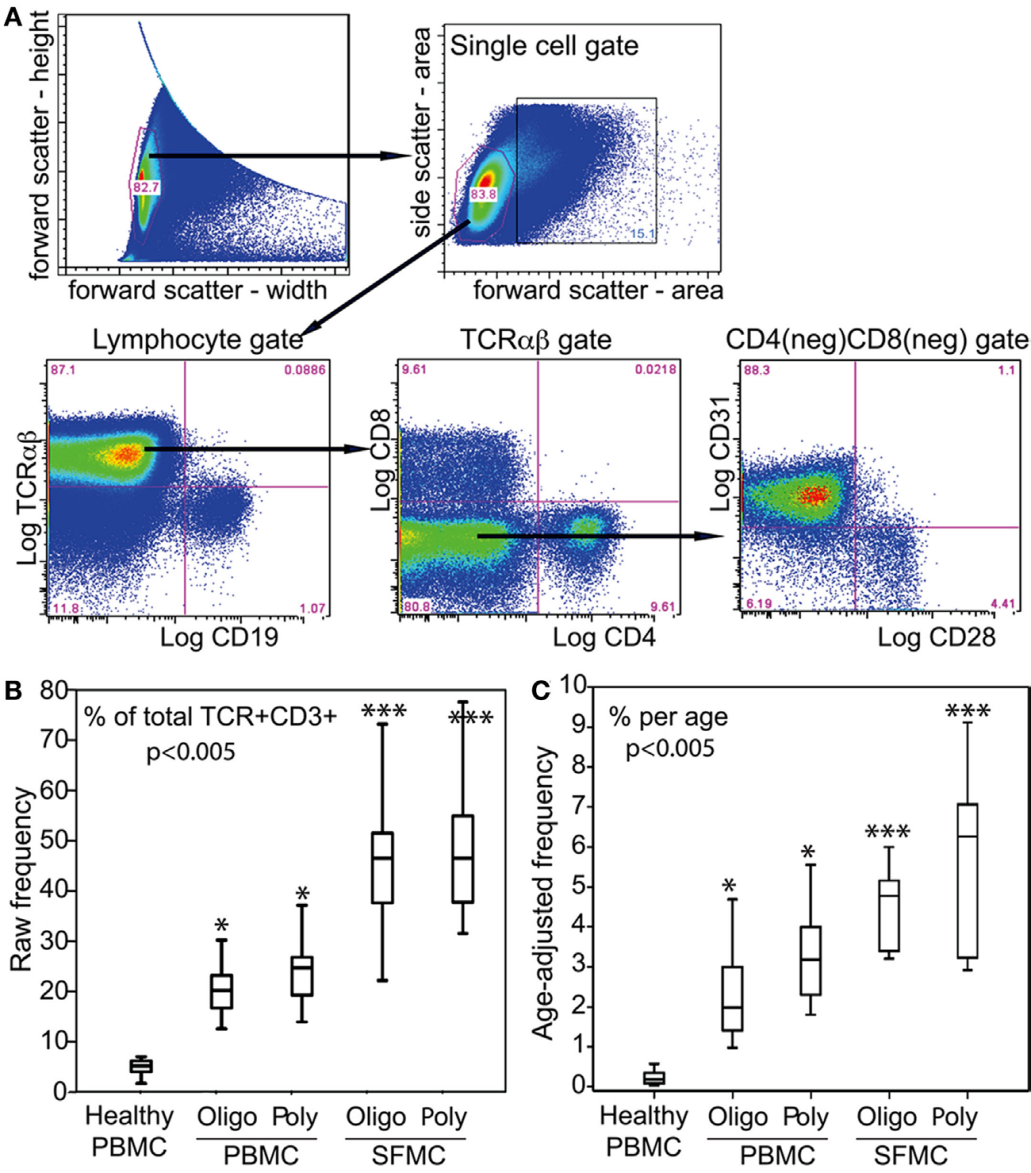
αβT cell profiles in PBMC and SFMC of JIA patients. **(A)** Illustrative flow of the electronic gating strategy for cytometric determination of the expression of TCRαβ, CD4, CD8, CD28, and CD31. The gating profile shown was for an SFMC sample, which was very similar with a parallel PBMC gating profile. Following an initial live/dead electronic gate, the height and width of the forward scatter was used to set a single cell gate for lymphocytes that were sequentially examined for CD4, CD8, CD28, and CD31. **(B)** Box-median-whisker plots shown are the raw frequency and **(C)** the age-adjusted frequency of CD31^+^CD28^null^DN αβT cells as a proportion of the total parent population of gated αβTCR^+^ cells. The plots were constructed as in Figure 1. The indicated *P*-values were determined by Kruskal–Wallis analysis of variance. *Post hoc* group comparisons by Tukey: ***P < 0.005 indicates SFMC of oligoarticular [oligo] and polyarticular [poly] JIA were significantly different from JIA/healthy PBMC; *P < 0.05 indicates JIA PBMC was significantly different from healthy PBMC.

We reported previously that JIA carry CD31^+^CD28^null^CD8^+^ T cells (7). Figure 2A illustrates the cytometry gating strategy for SFMC and PBMC. Focusing on TCRαβ^+^ gate, we found a new subset, namely, CD31^+^CD28^null^DN T cells. Figure 2B shows this subset comprised up to 80% (median ~48%) of the entire SF αβT cells in both oligoarticular and RF^−^ polyarticular JIA. The frequency of this SFMC subset was equivalent between the two patient groups. Using the same gating strategy for PBMC, the data also show similar CD31^+^CD28^null^DN T cells that constituted up to 38% of the total circulating αβT cells in oligoarticular and polyarticular JIA (medians of 21 and 26%, respectively). This DN T cell subset was found at very low frequency (<8%) in healthy PBMC.

CD28 is lost progressively with chronologic aging (8). Figure 2C shows that age-adjustment of the frequency of CD31^+^CD28^null^DN T cells reveal their annual accumulations for up to 5.3% per age-year in blood. The yearly medians between the two patient groups were equivalent, but these medians were significantly higher than the <0.8% accumulation per age-year in blood of healthy children. In SF, there were significantly higher accumulations of CD31^+^CD28^null^ DN T cells for up to 9.2% per age-year. The medians were 5 and 6.4% for oligoarticular and polyarticular JIA SFMC, respectively.

### CD31-Driven, TCR-Independent Expression of Cytokines, and Phosphorylation of Signaling Intermediates in Synovial CD31^+^CD28^null^ αβT Cells

**Figure 3:**
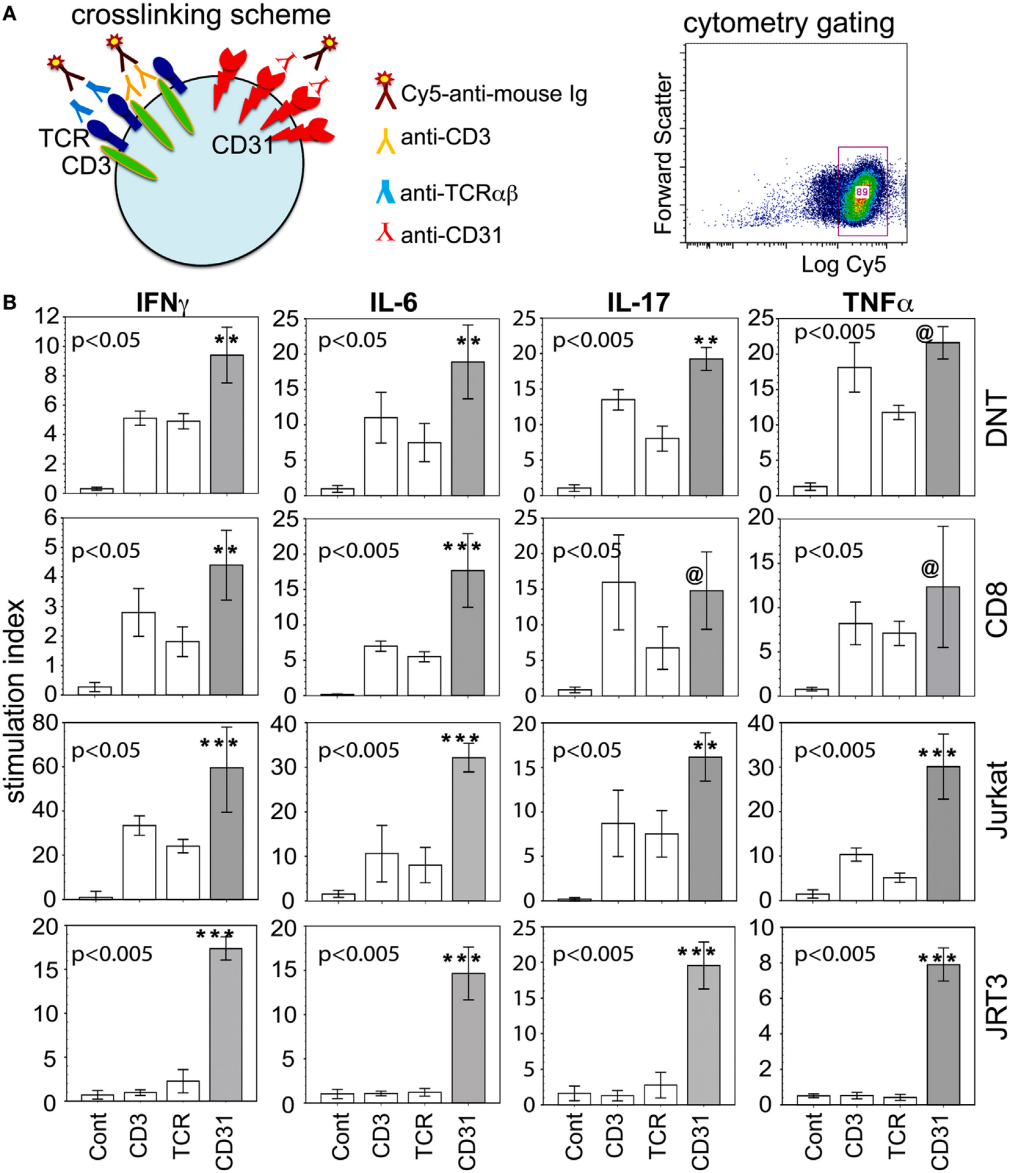
CD31 ligation alone is sufficient to induce intracellular expression of cytokines. **(A)** Diagram of receptor crosslinking with specific antibody, and the relevant gate of crosslinked (Cy5^+^) cells for subsequent cytometric analyses. **(B)** The data shown (bar means, SD whiskers; *n* = 5–11 per group) are intracellular levels of IFNγ, IL-6, IL-17, and TNFα in synovial CD31^+^CD28^null^ double negative (DN) and CD8^+^ αβT cells within 6 h of stimulation *via* CD3, TCRαβ, CD31, or IgG control. The data were expressed as stimulation index to normalize intrinsic variability between individual donors and culture batch differences of Jurkat or JRT3 cells. The plots were constructed as in Figure 1. The indicated *P*-values were determined by Kruskal–Wallis ANOVA. *Post hoc* group comparisons by Tukey: [***] and [**] *P* < 0.005 and *P* < 0.05, respectively, indicate CD31 crosslinking was significantly different from TCR or CD3 crosslinking and the IgG control [Cont] for synovial DN and CD8^+^ T cells, Jurkat, and JRT3; [@] indicates CD31 crosslinking was not significantly different from TCR and CD3 crosslinking, but was significantly different (*P* = 0.001) from the IgG control.

In keeping with our primary goal to more closely examine the microenvironment of the inflamed joint, bioassays were performed to determine whether synovial CD31^+^CD28^null^ subsets of DN and CD8^+^ αβT cells were sources of the cytokines detected in SF. Figure 3A illustrates the strategy for receptor crosslinking using specific antibodies to either CD31 (WM59), αβTCR (1P26), or CD3 (OKT3, Orthoclone), followed by Cy5-conjugated anti-mouse Ig. Cy5^+^ cells were then examined for the expression of CD8, CD4, CD31 (2H8), and intracellular cytokines. Figure 3B shows that within 6 h of CD31 ligation alone, there was induction of high levels of intracellular IL-6, IL-17A, IFNγ, and TNFα in primary CD31^+^ DN and CD8^+^ αβT cells. The levels of CD31-driven cytokine production were generally equivalent with those induced by CD3 or TCR ligation. For CD31^+^ DN T cells, CD31-induced production levels of IFNγ, IL-6, and IL-17A were significantly higher than the other stimulation groups. Similar higher levels of CD31-driven production of IL-6 and IFNγ were observed for CD31^+^ CD8^+^ T cells. Due to high staining background, cytoplasmic IL-10 was not measured.

To verify these results in a more tractable model, we examined CD3^+^TCR^+^CD31^+^ Jurkat. As depicted (Figure 3B third row), similar crosslinking of Jurkat recapitulated the cytokine production data from synovial CD31^+^ DN and CD8^+^ αβT cells. We also used JRT3, a somatic variant of Jurkat with mutated *CD3* and *TCRB* genes (ATCC) (36, 37), verified as CD3^−^ and αβTCR^−^, but CD31^+^, by cytometry. Consistent with these phenotypic characteristics, JRT3 cells showed an exclusive CD31-driven cellular expression of the same four cytokines (Figure 3B fourth row).

**Figure 4:**
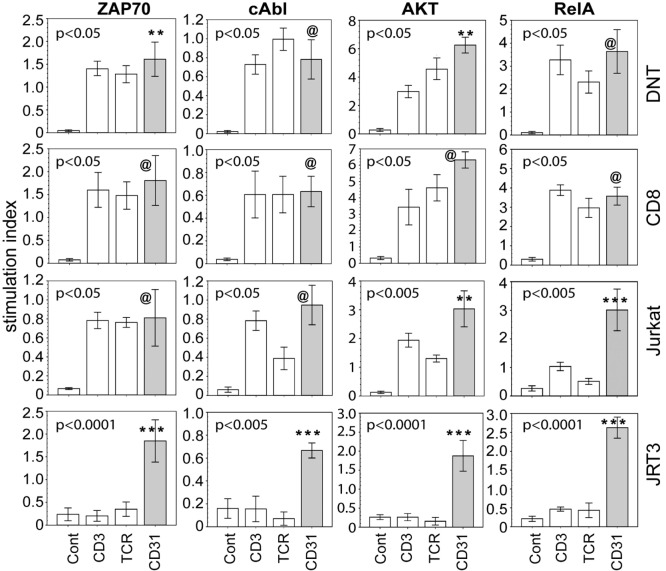
CD31 ligation alone is sufficient to elicit phosphorylation of signaling intermediates. Using the same crosslinking bioassay in Figure 3A, the data shown (bar means, SD whiskers; *n* = 5–9 per group) are phosphorylation levels of ZAP70, cAbl, AKT, and RelA within 15 min of stimulation *via* CD31, TCR, CD3, or IgG control. The data are normalized stimulation indices as in Figure 3B. The indicated *P*-values were determined by Kruskal–Wallis ANOVA. *Post hoc* group comparisons by Tukey: [***] and [**] *P* < 0.005 and *P* < 0.05, respectively, indicate CD31 crosslinking was significantly different from TCR or CD3 crosslinking or the IgG control [Cont]; [@] indicates CD31 crosslinking was not significantly different from TCR or CD3 crosslinking, but was significantly different (*P* = 0.0001) from the IgG control.

We also examined whether ligation of CD31 alone is sufficient to induce phosphorylation signaling intermediates. Specifically, we focused on ZAP70, Akt, RelA, and cAbl, four molecules found during an empirical screening of phosphoprotein expression following CD31 ligation (see Materials and Methods). ZAP70 and Akt are components of conventional TCR-driven activation of T cells (38, 39). RelA is a known component of the NFkB pathway linked to many inflammatory cascades (40). cAbl is of interest since it is not a known component of classical CD31 signaling, which has been studied extensively in non-immune cells (41, 42). Proving its mobilization following CD31 ligation on αβT cells would validate it as component of CD31-driven TCR-independent T cell-mediated inflammation. Figure 4 shows that within 10 min of CD31 ligation, there were highly significant phosphorylations of ZAP70, Akt, RelA, and cAbl compared to the IgG istotype controls in synovial DN and CD8^+^ αβT cells. The phosphorylation levels were largely equivalent between CD31, TCR, and CD3 ligations. In Jurkat, these phosphorylation events were reproduced in similar crosslinking assays. In JRT3, the phosphorylations were seen only in response to CD31 ligation.

### CD31-Driven cAbl Polarization, and Down Modulation of CD31-Driven Cytokine Production by an Inhibitor of cAbl and an Oxidoreductase Analog

**Figure 5:**
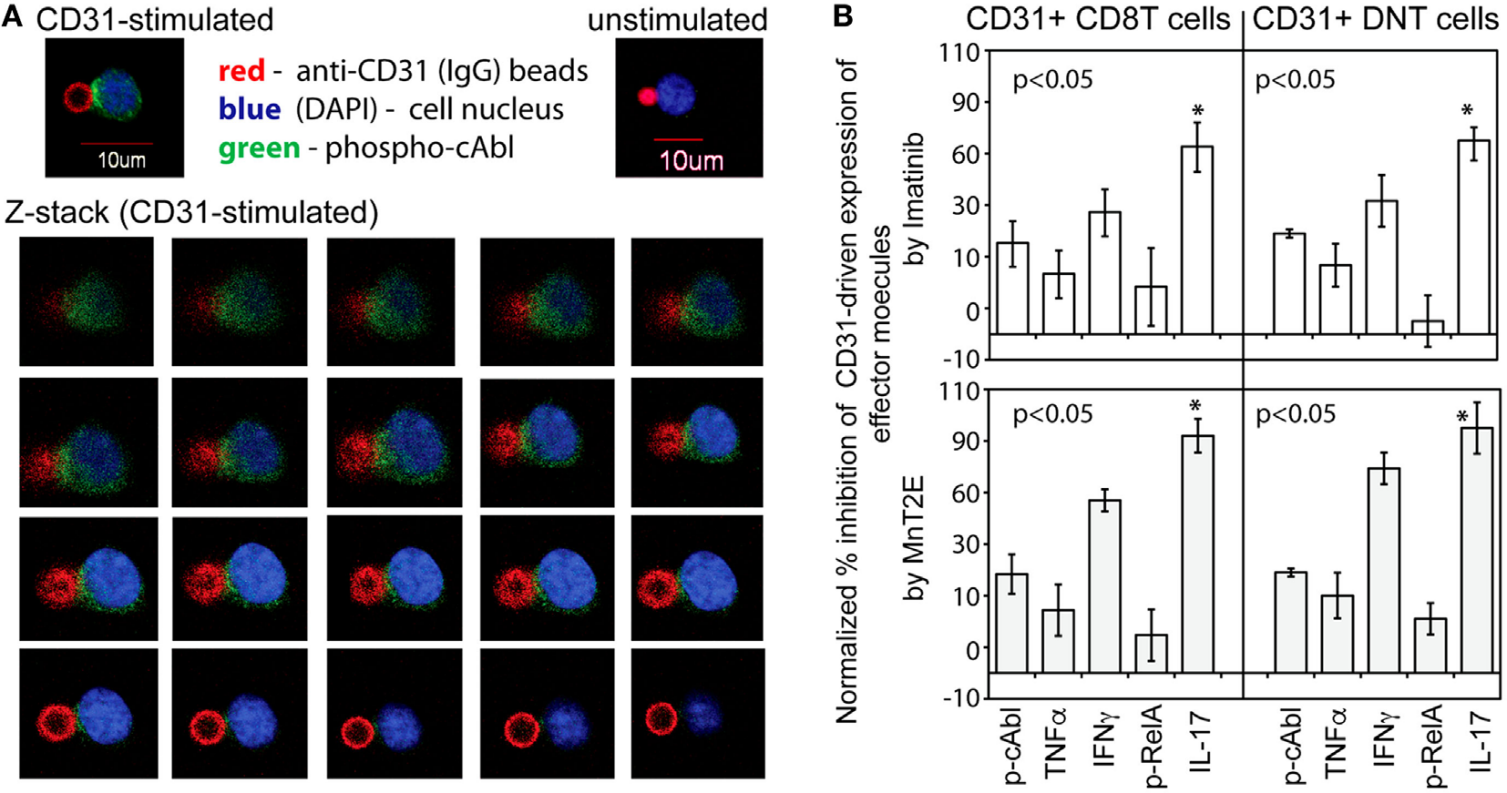
cAbl is a signaling substrate of CD31-driven TCR-independent activation of synovial αβT cells. **(A)** Micrographs shown are representative confocal images of CD31-driven polarization of cAbl in αβT cells from five independent experiments. The Z-stack (left to right order) were sequential image slices from the top to the bottom of a cell (DAPI and Green staining) and a fluorescent bead (Red) with immobilized anti-CD31. **(B)** The data shown (bar means, SD whiskers; *n* = 4–7 per group) are percent inhibition of CD31-driven expression of phospho-cAbl and phospho-RelA, and intracellular expression of TNFα, IFNγ, and IL-17A by 50 nM Imatinib or 34 µM MnT2E in CD31-crosslinked synovial CD31^+^CD28^null^ DN and CD8^+^ αβT cells CD31 crosslinking was performed as in Figure 3A. Data normalization was done as in Figures 3B and 4. The indicated *P*-values were determined by Kruskal–Wallis ANOVA. *Post hoc* group comparisons by Tukey: *P < 0.05 indicates MnT2E and Imatinib induced greater magnitudes of reduction of CD31-driven IL-17A expression than the inhibitor-induced reductions of cAbl, TNFα, IFNγ, and RelA expression.

Because classical CD31 signaling in non-immune cells (41, 42) has not been shown to involve cAbl, we analyzed whether cAbl was mobilized following TCR-independent CD31 ligation on T cells. Figure 5A shows representative imaging of phospho-cAbl polarization in five independent experiments. A Z-stack of confocal slices, and the single cell image (Inset, CD31-stimulated) showed phosphorylated cAbl localized at the point of contact between the T cell and the anti-CD31-bead. Polarized phospho-cAbl was observed at an average of 85% of cells per microscope field in 20 fields scanned in each of the five experiments. In contrast, cells incubated in IgG-bead had some random scattering of minutely speckled staining of cAbl, but there was no distinct polarization of the cAbl staining signal (Inset, Unstimulated). These observations indicated that CD31 signaling independent of TCR engagement in T cells involved cAbl.

The role of cAbl is further shown in Figure 5B. Imatinib, a known and clinically used inhibitor of constitutively active cAbl (22), consistently reduced the levels of CD31-driven phosphorylation of cAbl. In line with previous reports (43, 44), Imatinib also reduced the levels of RelA phosphorylation and intracellular expression of TNFα and IFNγ. Additionally, there was significant reduction of IL-17A expression. Similarly, MnT2E, a synthetic mimic of superoxide dismutase (23), significantly reduced CD31-driven RelA phosphorylation consistent with its reported inhibition of the DNA-binding activity of NFkB (24). MnT2E also reduced cAbl phosphorylation and intracellular expression of IL-17A, IFNγ, and TNFα. Unexpectedly, the level of suppression of CD31-driven expression of IL-17A by both Imatinib and MnT2E was significantly greater than the levels of suppression of IFNγ and TNFα expression.

### CD31-Driven, αβTCR-Independent Activation of *IL-17A* Gene Promoter

**Figure 6:**
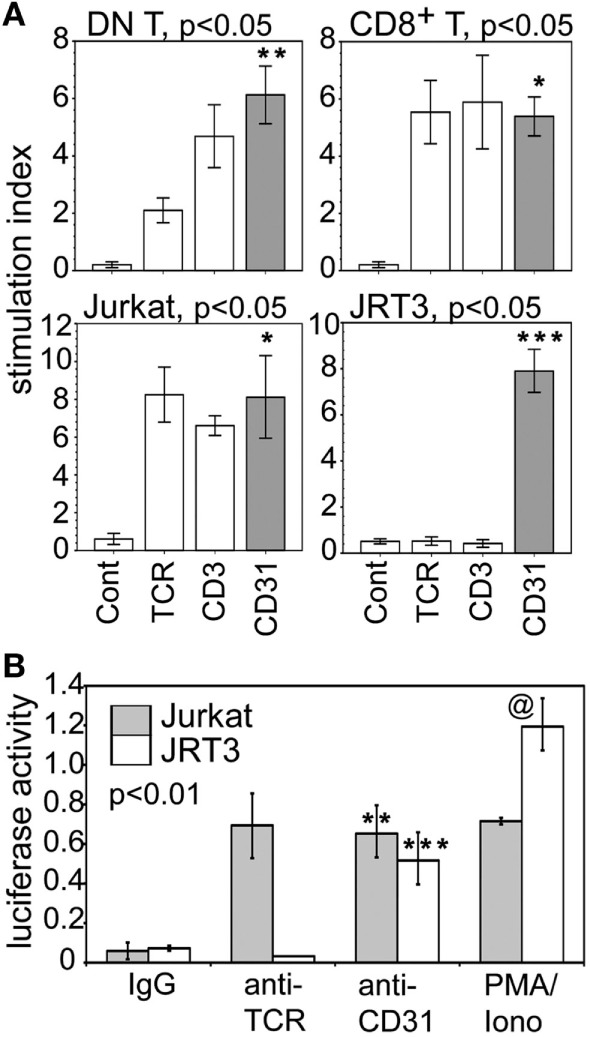
CD31-driven expression of RORγT and *trans*-activation of *IL-17A* gene promoter. **(A)** Data shown (bar means, SD whiskers; *n* = 5–7 per group) are RORγT levels in synovial CD31^+^CD28^null^ DN and CD8^+^ αβT cells, Jurkat, and JRT3 following CD31, TCR, CD3, or IgG stimulation. Crosslinking assays were performed as in Figure 3A. Data normalization done as in Figures 3B and 4. The indicated *P*-values were determined by Kruskal–Wallis ANOVA. *Post hoc* group comparisons by Tukey: ***P < 0.001 indicates CD31 crosslinking in JRT3 were significantly different from TCR and CD3 crosslinking, and the IgG control; **P < 0.05 indicates CD31 crosslinking on DN T cells were significantly different from TCR or CD3 crosslinking, or IgG control; * indicates crosslinking of CD31, TCR, or CD3 on CD8 T cells and Jurkat were not significantly different but were significantly different (*P* < 0.001) from the IgG control. **(B)** Data shown (mean bars, SD whiskers; *n* = 4–7 per group) are *IL-17A* gene promoter-driven luciferase reporter activities of Jurkat and JRT3. The data were normalized for transfection efficiency by co-transfection of *Renilla* luciferase plasmid. Receptor crosslinking was performed as in Figure 3A. The indicated *P*-value was determined by Kruskal–Wallis ANOVA. *Post hoc* group comparisons by Tukey: [**] indicates crosslinking of CD31 and TCR on Jurkat was not significantly different, but either one was significantly different (*P* < 0.005) than the IgG control; [***] *P* < 0.001 indicates CD31crosslinking on JRT3 cells were significantly different than TCR crosslinking or the IgG control; [@] *P* < 0.005 indicates control luciferase activity of phorbol myristyl acetate/Iono-stimulated JRT3 without receptor crosslinking was higher than Jurkat.

We further examined whether CD31 directly affected IL-17A production. Figure 6A shows CD31 ligation alone induced RORγT expression at significantly higher level than that induced conventionally *via* αβTCR or CD3 in synovial CD31^+^ DN and CD8^+^ αβT cells, and in Jurkat cells. For JRT3 cells, induction of RORγT expression was exclusive to CD31 ligation. Furthermore, Figure 6B shows CD31 ligation alone was sufficient to *trans*-activate the *IL-17A* gene promoter as assessed by luciferase reporter assays using Jurkat and JRT3. As expected, stimulation of Jurkat and JRT3 with the mitogen PMA/ionomycin, which bypasses TCR signaling, resulted in high luciferase activity compared to the IgG control albeit this JRT3 mitogenic response was higher than Jurkat.

### FLC Are Downstream Targets of IL-17A and Are Sensitive to Suppression by MnT2E as Effective as TNFi and IL6i

**Figure 7:**
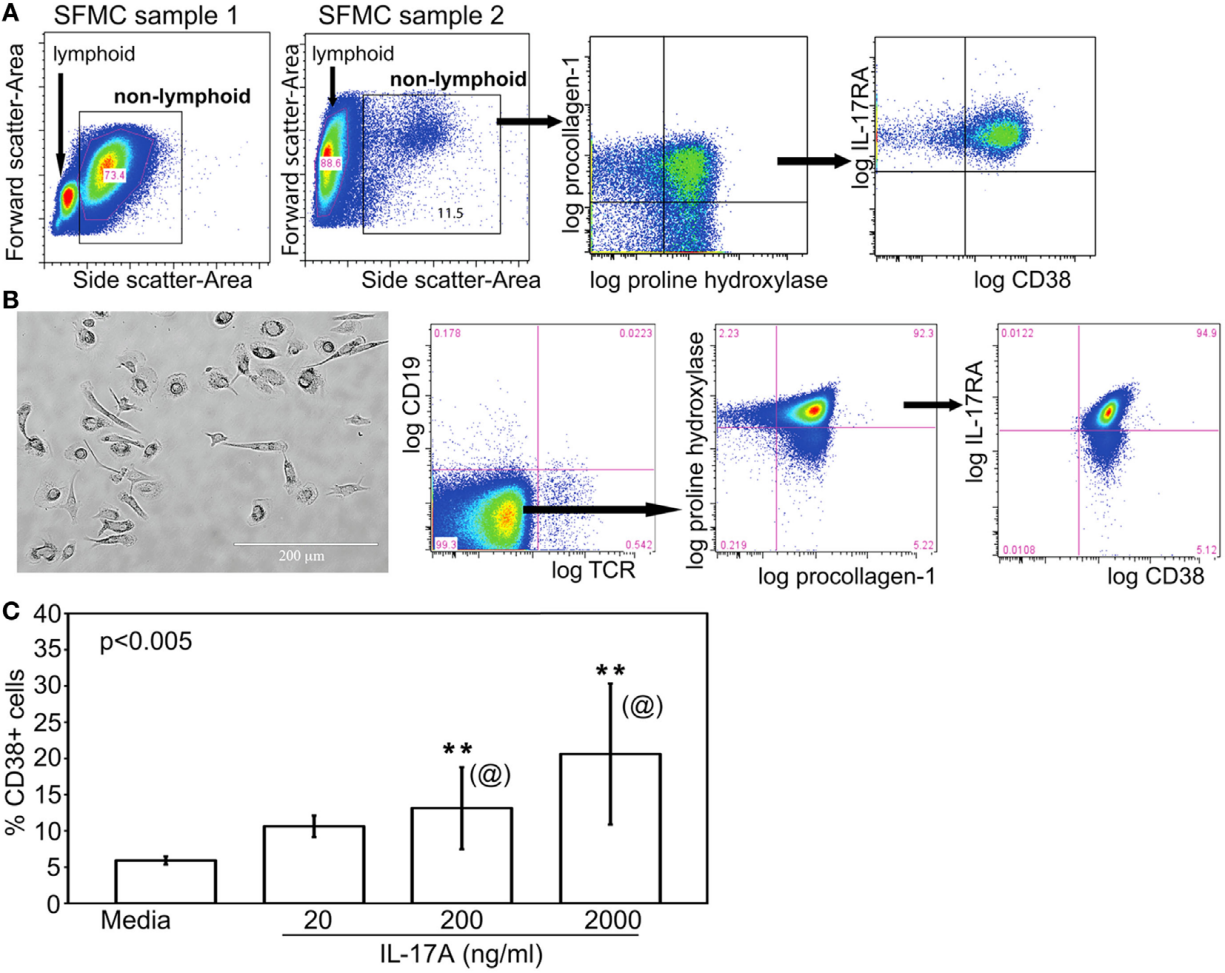
Fibrocyte-like cells are non-lymphoid IL-17A-responsive components of SF. **(A)** The general electronic gating strategy to set a live single cell gate was done as in Figure 2A. The cytograms shown illustrate the gated larger sized cells in SFMC, i.e., boxed higher forward versus side scatter. As depicted, the proportions of these non-lymphoid SFMC varied widely among patients. These cells co-expressed procollagen 1 and proline hydroxylase, known markers of fibroblasts and mesenchymal fibrocytes. These cells, referred to as FLC, also expressed IL-17RA and CD38. **(B)** Representative micrograph of 10–15 day cultured, plate-adherent FLC showing stellate to ameboid morphology. Their typical cytogram profile showed negative staining for T and B cell markers, but positive staining of proline hydroxylase, procollagen 1, IL-17RA, and CD38. **(C)** CD38 expression on FLC (bar means, SD whiskers; *n* = 5 per group) incubated with three doses of recombinant IL-17A for 24 h. The indicated *P*-value was determined by Kruskal–Wallis ANOVA. *Post hoc* group comparisons by Tukey: [@] indicate no significant differences between 200 and 2,000 ng/ml doses of IL-17A; [**] indicates responses to 200 or 2,000 ng/ml was significantly different (*P* < 0.05) than those seen with 20 ng/ml IL-17A or media control.

In cytometric analyses of SFMC, we routinely noticed the presence of SFMC of larger size (forward scatter) and higher granularity (side scatter) compared to the electronically gated lymphocytes (depicted by bounding box in Figure 2A). Figure 7A illustrates individual variations in the proportions of these non-lymphoid SFMC, which were recognized from the exclusion staining of TCRαβ, TCRγδ, CD3, CD4, CD16, CD19, and CD56 by back-gating strategies. These non-lymphoid cells uniformly co-expressed procollagen 1 and proline-4-hydroxylase, the typical markers of fibroblasts and circulating fibrocytes (18–20). They also expressed the receptor for IL-17 (IL-17RA) and CD38, a ligand for CD31 (45). Further, Figure 7B shows these non-lymphoid cells were adherent to plastic and were amenable for short-term propagation. They had varying stellate to ameboid morphology, and retained expression of procollagen 1, proline-4-hydroxylase, IL-17RA, and CD38. These morphological characteristics underscore their designation as “fibrocyte-like cells.”

It is not yet known whether FLC in JIA SFMC derive from the same lineage as the hyperplastic fibroblast-like synoviocytes found in adult RA synovial tissue, which may be similarly positive for procollagen 1 and proline-4-hydroxylase (46). It is also unclear if FLC were fibrocytes, which could be found in small numbers in blood (18) and then infiltrated the joint. Neither is yet known if FLC were fibroblasts that detached from the pannus of the inflamed JIA joint. Irrespective of their origin, Figure 7C shows that consistent with their expression of IL-17RA, FLC exposed to recombinant IL-17A showed a dose-dependent increased expression of CD38. There were no differences in phenotype and responsiveness to IL-17A of FLC between oligoarticular and RF^−^ polyarticular JIA.

**Figure 8:**
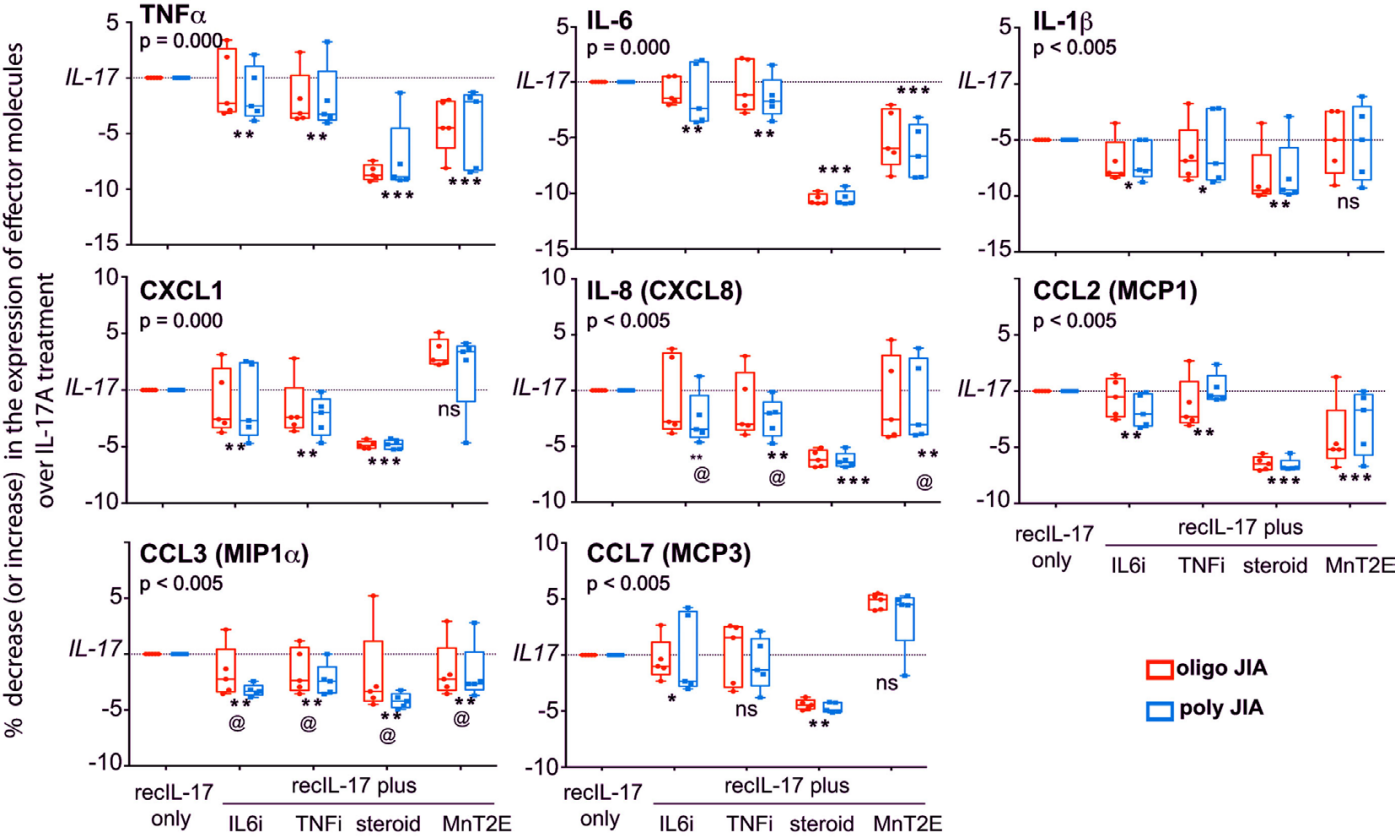
IL-17A-mediated production of cytokines and chemokines by FLC, and their sensitivity to MnT2E. Data shown are box-median-whisker plots (*n* = 5–7 per group), which were constructed as in Figure 1. As depicted, the maximum levels of production of each of the indicated molecules by FLC cultures in 200 ng/ml recombinant IL-17A [recIL-17] were set as 100% response. Production levels of each indicated molecular effector in FLC cultures with recIL-17 combined with 5 µM corticosteroid or IL6i or TNFi, or 34 µM MnT2E were normalized as percent increase or decrease over the maximal response to recIL-17. The indicated *P*-values were determined by Kruskal–Wallis ANOVA. *Post hoc* group comparisons by Tukey: ***P < 0.005 indicate corticosteroid- and/or MnT2E-mediated suppression of IL-17A-mediated production of effectors was significantly greater than those elicited by TNFi or IL6i; [**] and [*]*P* < 0.01 and *P* < 0.05, respectively, indicate significant suppression of production of effector molecules compared recIL17 only group; [@] no significant differences among inhibitor-treated groups, but significantly different (*P* < 0.05) from the recIL-17 only group; ns, not significantly different between the inhibitor-treated group(s) and recIL-17 only group.

**Figure 9:**
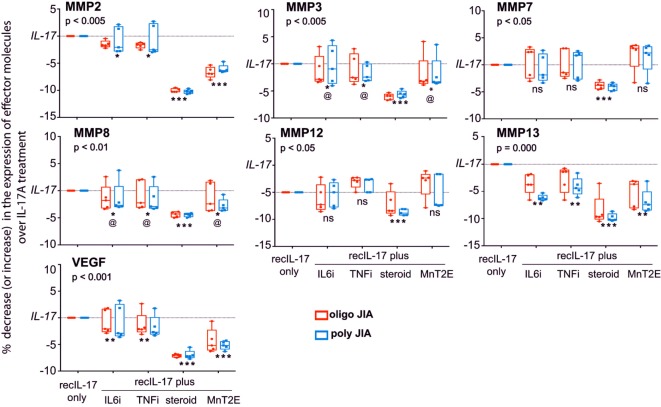
Sensitivity of IL-17A-mediated production of tissue-destructive proteins by FLC to down modulation by MnT2E. Culture supernatants from the same FLC bioassays in Figure 8 were also examined for MMPs and vascular endothelial growth factor (VEGF). Sample sizes, box-median-whisker plots, and statistical analyses were identical to that of Figure 8.

The responsiveness of FLC to IL-17A was further examined by the addition of MnT2E, the biologics of TNFi and IL6i, or corticosteroid to the bioassays. Here, we focused on IL-17A-induced molecular effectors from experimental systems including those implicated in juvenile and adult arthritis (21, 26–29). Out of 18 molecular effectors examined, 3 cytokines (TNFα, IL-6, and IL-1β) and 5 chemokines [CXCL1, CXCL8 (IL-8), CCL2 (MCP1), CCL3 (MIP1α), and CCL7 (MCP3)] were found to be significantly induced IL-17A (Figure 8). Additionally, IL-17A induced production of tissue-destructive proteins, namely, 6 metalloproteinases (MMP; MMP2, 3, 7, 8, 13, and 13) and vascular endothelial growth factor (VEGF) (Figure 9). There were no significant differences in MMP1, MMP9, and tissue inhibitor of metalloproteinase-1 levels between control FLC cultures and those incubated in IL-17A. Production of the 15 IL-17A-induced effectors by FLC was uniformly down-regulated by corticosteroid. Similarly, IL6i and TNFi down-regulated the expression of these effectors, albeit at lower magnitudes compared to those seen with corticosteroid.

The data also show that MnT2E was capable of inhibiting IL-17A-induced production of inflammatory effectors by FLC. Its inhibitory effects were not as high as those seen with corticosteroid. However, MnT2E was as effective as IL6i and TNFi in reducing IL-17A-mediated production of MMP3, MMP8, and IL8 (CXCL8). It elicited significantly higher degrees of inhibition of IL-17A-mediated production of TNFα, IL-6, MMP2, VEGF, and CCL2 (MCP1) than either IL6i or TNFi. MnT2E, IL6i, and TNFi did not affect the production of MMP7 and MMP12. MnT2E also did not affect production of IL-1β. Both TNFi and MnT2E had no effect on CCL7 (MCP3) production. Generally, there were no significant differences in FLC responses between oligoarticular and RF^−^ polyarticular JIA.

## Discussion

Cytokines/chemokines are considered pathologic effectors of JIA. Their plasma/serum levels have been variably associated with disease activity, particular disease manifestations, or responses to biologics such as IL-6i and TNFi (47, 48). Regardless of treatment, many patients experience episodes of arthritic flares that are usually managed by systemic and/or by local therapy with arthrocentesis and corticosteroid injection. During such flares, there is exaggerated synovial inflammation compared to blood that is reflected by non-correspondence between plasma/serum and SF cytokine profiles (21). Thus, biological analysis of SF is a preferable approach to better understand the nature of synovitis in JIA (49).

The present study shows variable and low levels of plasma cytokines, some of which are not significantly different between JIA and healthy controls. In contrast, the SF cytokine profiles show dominance of IL-6, IL-10, IL-17A, IFNγ, and TNFα, which were among those identified by de Jager et al. (21). By comparison, our data show 4–5 orders of magnitudes for these five molecules (present data in Figure 1 in ng/ml quantities versus data **Table 3** of de Jager et al. in pg/ml). Such quantitative differences could be related to intrinsic cohort differences. IL-6 and TNFα are two of the most consistently reported cytokines in JIA (21, 47). IL-10 has both pro- and anti-inflammatory effects in human disease and is among the upregulated cytokines in clinically active JIA (50). IFNγ has been associated with innate and adaptive responses in adult RA, with some reported association with systemic-onset JIA but not with oligoarticular or RF^−^ polyarticular JIA (51). IL-17A is a cytokine of interest in the biology of JIA (52). As shown by the present data and those reported by de Jager et al. (21), there are equivalent levels of these five cytokines in SF of oligoarticular and RF^−^ polyarticular JIA. Collectively, these findings support a growing opinion in Pediatric Rheumatology that these two clinical subtypes may represent a continuum of the same disease (14). Inasmuch as the present study is a cross section of patients with long-standing disease (disease duration up to 15 years) that have a treatment history with various medications, the dominance of IL-6, IL-10, IL-17A, IFNγ, and TNFα in SF suggests a common cytokine signature of JIA synovitis.

A key question is whether the SF cytokine milieu is linked to discrete subset(s) of joint-infiltrating cells. The present study provides evidence for the role of DN αβT cells. Whether these cells come from a distinct lineage or are derived from chronic inflammatory activation of single-positive CD4 and/or CD8 precursors remains to be examined. However, expression levels of CD4 and CD8 have been known to be transiently downregulated during conventional TCR-crosslinking (53). In T cell cultures with highly mitogenic stimuli such as anti-CD3/CD28 or phorbol ester/ionomycin, extremely high rates of proliferation of CD8, but not CD4, cells were reportedly associated with the emergence of DN T cells (54). Such *in vitro* emergence of DN T cells has been linked to epigenetic modification of *CD8* that renders it inaccessible to transcription (55). Along these lines, DN T cells have also been reported to constitute ~10% of blood T cells in adults with systemic lupus erythematosus (56), a disease with known global and *CD8*-specific epigenetic modifications (57). Similar epigenetic modifications have been reported for CD4^+^ T cells in JIA (58), but it is not yet clear if such changes lead to conversion of single-positive CD4 into DN T cells. Whether epigenetics regulate lineage decisions of joint-infiltrating T cells in manner differently from that of T cells residing/transiting in normal lymphoid tissue is also unknown. Regardless of the role of epigenetics, we report here that compared to adult lupus, oligoarticular and RF^−^ polyarticular JIA have greater than twice the frequency of DN T cells in blood, and up to sevenfold higher in SF.

Our new finding is that DN αβT cells in JIA are CD31^+^CD28^null^, a phenotype reminiscent of a CD8^+^ subset we reported previously (7). This is unlike the situation in adult lupus where DN T cells are CD28^+^ (56) and CD31 expression has not been examined. CD28^null^ T cells are a biomarker of normal aging or premature aging in the human immune system (10). We have shown that the irreversible loss of CD28 can be accelerated by persistent TCR stimulation, and by inflammatory mediators (32, 59). As for CD31, it is a known marker for fresh naïve CD28^+^CD4^+^ T cells and is lost when they become CD28^+^ activated or CD28^null^ memory CD4 effectors (60). We have shown that CD31 is sporadically expressed on fresh naïve CD8^+^ T cells, but is stably expressed on highly activated CD8 cells, and upon their conversion from CD28^+^ to memory CD28^null^CD8^+^ effectors (7). Whether the losses of CD4, CD8, and CD28, and the corresponding gain of CD31 are independent, co-dependent, or sequential events remain to be examined.

The abundance of CD31^+^CD28^null^ DN and CD8^+^ αβT cells in SF suggests their pathogenic role. Our data show >2.5-fold annual increase in CD31^+^CD28^null^DN αβT cell frequency in SF compared to that seen in blood. Such yearly accumulation is reminiscent of our original report for CD31^+^CD28^null^CD8^+^ αβT cells (7). However, a longitudinal analysis of paired blood and SF samples is needed to ascertain whether there is concordance of their cell frequencies increases over time. Longitudinal studies could also inform whether arthritic flares may be due to, or predicted by cumulative increases in, CD31^+^CD28^null^ DN and/or CD8^+^ αβT cells, or to particular effector subsets thereof.

An experimental support for a pathogenic role for CD31^+^CD28^null^ DN and CD8^+^ αβT cells is their TCR-independent activation. Our data show CD31 ligation alone sufficiently increase intracellular IL-6, IL-17A, IFNγ, and TNFα, four of the five most upregulated cytokines we found from SF cytokine profiling. Furthermore, there is CD31-driven, TCR-independent phosphorylation of ZAP70, Akt, and RelA, three components of conventional TCR-driven CD28-dependent T cell signaling (38–40). Our data also show a primary role of cAbl in CD31-driven activation of synovial T cell activation; cAbl is not a known component of CD31 signaling in non-immune cells (41, 42). For conventional αβT cells, cAbl has been implicated as a minor secondary participant of TCR/CD28 signaling (61). Our data indicate cAbl along with ZAP70, Akt, and RelA are proximal transducers of CD31 signaling. Whether these signaling molecules are independently or co-dependently required for the CD31-driven production of distal cytokine effectors, such as IL-6, IL-17A, IFNγ, and TNFα by αβT cells remains to be examined. Nonetheless, our data sets clearly show CD31 signaling in synovial αβT cells effectively suborn or co-opt the classical TCR signaling pathway in the absence of TCR engagement. This idea is supported by our data set with TCR^+^CD3^+^CD31^+^ Jurkat and TCR^−^CD3^−^CD31^+^ JRT3 that recapitulated the CD31-driven cytokine production and the phosphorylations of signaling intermediates. Given the basic tenet that antigen-specific TCR triggering confers protective immunity, CD31-driven TCR-independent activation of synovial CD31^+^ DN and CD8^+^ αβT cells may represent a form of immune dysregulation that contribute to JIA synovitis.

IL-17A is a cytokine of interest in many chronic inflammatory diseases (26, 52, 62, 63). We report here several novel findings. First, our data provide evidence for TCR-independent IL-17A production by synovial DN and CD8^+^ αβT cells. CD31 ligation alone is sufficient to induce expression of RORγT transcription factor and *trans*-activation of *IL-17A* gene promoter. Thus, in addition to IL-17A^+^ CD4^+^ αβT and γδT cells (52, 64, 65), CD31^+^CD28^null^ DN and CD8^+^ αβT cells likely represent non-conventional Th17 subsets. Whether all these IL-17A-producing subsets of T cells work in concert or have differential roles during arthritic flares in JIA has yet to be examined.

Second, CD31-driven IL-17A production is suppressed by Imatinib, an anti-CML drug known to inhibit cAbl kinase activity (22). The signaling pathway linking CD31 ligation, cAbl phosphorylation, and IL-17A production needs further investigation.

Third, a target of IL-17A in the joint is IL-17RA^+^ FLC. Their exposure to IL-17A results in the production of several MMPs, chemokines, and VEGF, and additional TNFα and IL-6. FLC also express the CD31 ligand CD38 (45). We validated previously that recombinant CD38-Ig stimulates CD31^+^CD28^null^CD8^+^ αβT cells (7). The origin(s) of FLC has yet to be determined.

Fourth, CD31-driven production of IL-17A by CD31^+^CD28^null^ DN and CD8^+^ αβT cells is sensitive to MnT2E, a mimic of superoxide dismutase (23). The suppressive activity of MnT2E on synovial αβT cell cytokine production is in line with its reported down modulatory effect on T cells in non-obese diabetic mice wherein oxyradicals play a significant role in insulitis and pancreas pathology (66). Our data showing MnT2E-mediated inhibition of RelA phosphorylation in synovial CD31^+^ DN and CD8^+^ T cells is also consistent with its reported blockade of the DNA-binding activity of NFκB in whole tissue explants (24). Furthermore, our data show MnT2E-dependent inhibition of cAbl phosphorylation, and a higher degree of MnT2E inhibition of CD31-driven production of IL-17A. These two particular suppressive effects MnT2E are equivalent to that seen with Imatinib. These suggest Imatinib and MnT2E may be useful tools to further probe CD31→cAbl→IL-17A directional signaling.

It remains to be investigated whether MnT2E directly affects structure or function of cAbl. However, c-Abl has been shown experimentally to be modulated to reactive oxygen species (67). This could explain our observation that c-Abl phosphorylation was indeed affected by MnT2E consistent with its known dismutase activity (23). It also remains to be examined whether MnT2E directly alters the transcriptional and/or translational machineries of IL-17A production. This is of interest since the *IL-17A* gene promoter includes an NFκB binding site (30). RelA complexes of NFκB are known targets of oxyradicals (68). IL-17A production itself appears to be sensitive to redox reactions (69).

Finally, MnT2E also affects IL-17A-mediated production of additional inflammatory effectors by FLC. MnT2E suppression of MMP3, MMP8, and IL-8 (CXCL8) expression is comparable to those seen with IL6i and TNFi. MnT2E produces greater degrees of inhibition TNFα, IL-6, MMP2, VEGF, and MCP1 (CCL2) production than either IL6i or TNFi. MnT2E, however, has no effect on MMP7, MMP12, and IL-1β. The basis for these differential effects of MnT2E has to be examined.

**Figure 10:**
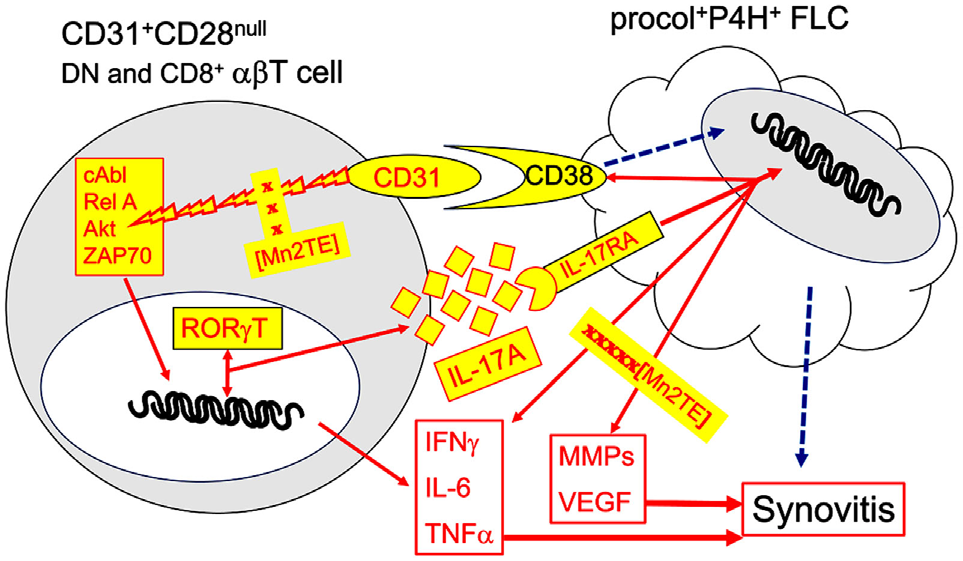
Model for an inflammatory circuit between CD31^+^CD28^null^ αβT cells and FLC in JIA synovitis. The present data (Figures 2 and 7) authenticate enrichment of CD31^+^CD28^null^ DN αβT cells, as well as procollagen [procol]^+^proline hydroxylase [P4H]^+^ FLC in SF of oligoarticular and rheumatoid factor^−^ polyarticular JIA. In previous work, we have shown a similar CD31^+^CD28^null^ subset in the CD8^+^ compartment (7). Ligation of CD31 with specific antibody on these two αβT cell subsets sufficiently and effectively signals phosphorylation of ZAP70, Akt, cAbl, and RelA (Figure 4). It is also sufficient to induce production of IL-17A, IL-6, IFNγ, and TNFα (Figure 3). These four cytokines are among the most dominant cytokines identified in SF (Figure 1). We have shown previously that similar cytokine production is achieved by CD31 ligation with CD38-Ig (7), indicating CD38 is a true ligand, CD31 ligand on synovial CD31^+^ αβT cells. Relevance of CD31 as a driver IL-17A is indicated by the specific *trans*-activation of *IL-17A* promoter and induction RORγT (Figure 6). CD31-driven production of cytokines and phosphorylation of cAbl and RelA are sensitive to down regulation by Imatinib, a known cAbl inhibitor, and by MnT2E, a superoxide mimic, and inhibitor of the DNA-binding activity of NFκB (Figure 5). IL-17RA^+^CD38^+^ FLC is a likely target of IL-17A as indicated by the upregulation of CD38 (Figure 7). Futhermore, IL-17A induces FLC to produce additional inflammatory cytokines/chemokines and tissue-destructive effectors, which are sensitive to down modulation by MnT2E (Figures 8 and 9). Such inhibitory activity of MnT2E on FLC is comparable, and in some cases, better than the biologics IL6i and TNFi. Details of IL-17A and CD38 signaling in FLC [dashed arrows] remain to be examined.

In summary, the present work provides evidence for TCR-independent, CD31-driven activation of joint-infiltrating CD31^+^CD28^null^ DN and CD8^+^ αβT cells in JIA. These cells are sources of IL-6, IL-17A, IFNγ, and TNFα, four abundant cytokines in SF. A downstream target of IL-17A is IL17RA^+^CD38^+^ FLC, which responds by further upregulating CD38, a ligand of CD31 (45), and the production of additional inflammatory and tissue-destructive effectors including TNFα and IL-6 that compounds the CD31-driven cytokine production by αβT cells. Collectively, our data suggest a synovial T cell-FLC inflammatory circuit illustrated in Figure 10. The working model highlights plausible CD31–CD38 interaction with an IL-17A-mediated feedback loop. We have shown previously (7) that CD38-Ig is potent inducer of cytokine production by CD31^+^CD28^null^ αβT cells. Specificity of a CD31–CD38 cognate interaction will have to be verified. Blocking CD31 and/or CD38 in usual T cell-FLC co-cultures yielded very variable results, which were likely due to a variety of compounding receptor-counter receptor interactions. Nuances of IL-17A-signaling and CD38-signaling cascades in FLC also remain to be examined. Admittedly, the model cannot exclude other possible feedback loops, since CD31-activated T cells and IL-17A-activated FLC produce multiple effectors. Nonetheless, a notable aspect of our model is the identification of MnT2E as an independent inhibitor of CD31-driven T cell activation and IL-17-A-mediated FLC activation. This points to its likely utility as a probe to further unravel intricacies of CD31 signaling in synovial αβT cells, as well as IL-17A and CD38-signaling cascades in FLC. The inhibitory activity of MnT2E also provides a translational rationale to test whether it is potentially disease-modifying.

## Ethics Statement

Institutional Review Boards of the University of Pittsburgh approved all research protocols. Written informed consent from legal guardians, and assent of child subjects as appropriate, were obtained.

## Author Contributions

AV designed the study and secured funding. IF, PG, JM, and AD prepared the manuscript. AV, JM, and DK designed and managed the IRB protocol. IF, JM, JD, MR, and DK recruited/consented subjects and abstracted medical records. IF, PG, JM, RM, and JD collected, processed, and cataloged biological specimens. IF, PG, HY, RM, and JD performed experiments. SG and JP provided critical reagents and designed their use. IF, PG, JM, and AV performed statistical analysis. All authors reviewed/approved the manuscript.

## Conflict of Interest Statement

All authors have no financial conflict of interest. JP is co-owner of a patent (US Patent Application #20030032634) on the oxidoreductase analog MnT2E used in this study. Albany Molecular Research Institute Inc., (AMRI), a company licensed for clinical testing of MnT2E, provided this agent for research but was not involved in funding, design, implementation, or publication of this study. None of the authors have any relationship with AMRI.

## Abbreviations (non-standard Immunology)

ANA, anti-nuclear antibody; DN, double negative; FLC, fibrocyte-like cells; IL6i, clinically used biologic inhibitor of IL-6; JIA, juvenile idiopathic arthritis; MnT2E, Mn(III) meso-tetrakis(N-ethylpyridinium-2-yl)porphyrin; RA, rheumatoid arthritis; RF, rheumatoid factor; SF, synovial fluid; SFMC, mononuclear cells in SF; TNFi, clinically used biologic inhibitor of TNF.
